# Insulin Induces Relaxation and Decreases Hydrogen Peroxide-Induced Vasoconstriction in Human Placental Vascular Bed in a Mechanism Mediated by Calcium-Activated Potassium Channels and L-Arginine/Nitric Oxide Pathways

**DOI:** 10.3389/fphys.2016.00529

**Published:** 2016-11-22

**Authors:** Lissette Cabrera, Andrea Saavedra, Susana Rojas, Marcela Cid, Cristina Valenzuela, David Gallegos, Pamela Careaga, Emerita Basualto, Astrid Haensgen, Eduardo Peña, Coralia Rivas, Juan Carlos Vera, Victoria Gallardo, Leandro Zúñiga, Carlos Escudero, Luis Sobrevia, Mark Wareing, Marcelo González

**Affiliations:** ^1^Vascular Physiology Laboratory, Department of Physiology, Faculty of Biological Sciences, Universidad de ConcepciónConcepción, Chile; ^2^Department of Morphophysiology, Faculty of Medicine, Universidad Diego PortalesSantiago, Chile; ^3^Department of Obstetrics and Childcare, Faculty of Medicine, Universidad de ConcepciónConcepción, Chile; ^4^Department of Pathophysiology, Faculty of Biological Sciences, Universidad de ConcepciónConcepción, Chile; ^5^Group of Research and Innovation in Vascular Health (GRIVAS Health)Chillán, Chile; ^6^Centro de Investigaciones Médicas (CIM), School of Medicine, Universidad de TalcaTalca, Chile; ^7^Vascular Physiology Laboratory, Group of Investigation in Tumor Angiogenesis (GIANT), Department of Basic Sciences, Universidad del BiobíoChillán, Chile; ^8^Cellular and Molecular Physiology Laboratory (CMPL), Division of Obstetrics and Gynecology, School of Medicine, Faculty of Medicine, Pontificia Universidad Católica de ChileSantiago, Chile; ^9^Department of Physiology, Faculty of Pharmacy, Universidad de SevillaSeville, Spain; ^10^University of Queensland Centre for Clinical Research (UQCCR), Faculty of Medicine and Biomedical Sciences, University of QueenslandHerston, QLD, Australia; ^11^Maternal and Fetal Health Research Centre, Institute of Human Development, University of ManchesterManchester, UK; ^12^Maternal and Fetal Health Research Centre, St. Mary's Hospital, Central Manchester University Hospitals NHS Foundation Trust, Manchester Academic Health Science CentreManchester, UK

**Keywords:** insulin, L-arginine, nitric oxide, hCAT-1, BKCa channels, placenta

## Abstract

**HIGHLIGHTS**

- Short-term incubation with insulin increases the L-arginine transport in HUVECs.
- Short-term incubation with insulin increases the NO synthesis in HUVECs.
- Insulin induces relaxation in human placental vascular bed.
- Insulin attenuates the constriction induced by hydrogen peroxide in human placenta.
- The relaxation induced by insulin is dependent on BKCa channels activity in human placenta.

Insulin induces relaxation in umbilical veins, increasing the expression of human amino acid transporter 1 (hCAT-1) and nitric oxide synthesis (NO) in human umbilical vein endothelial cells (HUVECs). Short-term effects of insulin on vasculature have been reported in healthy subjects and cell cultures; however, its mechanisms remain unknown. The aim of this study was to characterize the effect of acute incubation with insulin on the regulation of vascular tone of placental vasculature. HUVECs and chorionic vein rings were isolated from normal pregnancies. The effect of insulin on NO synthesis, L-arginine transport, and hCAT-1 abundance was measured in HUVECs. Isometric tension induced by U46619 (thromboxane A_2_ analog) or hydrogen peroxide (H_2_O_2_) were measured in vessels previously incubated 30 min with insulin and/or the following pharmacological inhibitors: tetraethylammonium (KCa channels), iberiotoxin (BKCa channels), genistein (tyrosine kinases), and wortmannin (phosphatidylinositol 3-kinase). Insulin increases L-arginine transport and NO synthesis in HUVECs. In the placenta, this hormone caused relaxation of the chorionic vein, and reduced perfusion pressure in placental cotyledons. In vessels pre-incubated with insulin, the constriction evoked by H_2_O_2_ and U46619 was attenuated and the effect on H_2_O_2_-induced constriction was blocked with tetraethylammonium and iberiotoxin, but not with genistein, or wortmannin. Insulin rapidly dilates the placental vasculature through a mechanism involving activity of BKCa channels and L-arginine/NO pathway in endothelial cells. This phenomenon is related to quick increases of hCAT-1 abundance and higher capacity of endothelial cells to take up L-arginine and generate NO.

## Introduction

In the human placenta, an organ lacking innervation, endothelial factors released in response to shear stress, oxygen level, paracrine, or endocrine signals, are the main mechanisms that maintain low resistance and blood flow in the placental vascular bed (Wareing, 2014). In this regard, insulin induces relaxation in human umbilical veins via a mechanism involving increased transcriptional activity of *SLC7A1* (coding for human Cationic Amino Acid Transporter-1, hCAT-1) and L-arginine transport (González et al., 2011). In addition, insulin also increases nitric oxide (NO) synthesis through activation of phosphatidylinositol 3-kinase (PI3K) and endothelial NO synthase (eNOS) in HUVECs (González et al., 2004). We reported that insulin may also generate hyperpolarization in this cell type (González et al., 2004), which enhances vasomotor activity of this hormone in the placental macrocirculation and microcirculation. Despite this evidence, detailed mechanisms underlying the vasomotor activity of insulin are still unclear.

Regulation of the plasma membrane potential in both endothelial cells and vascular smooth muscle cells (VSMCs) involves potassium (K^+^) channel activity—dependent hyperpolarization (Durand and Gutterman, 2013). The human placenta expresses large conductance calcium-activated K^+^ channels (BKCa) (Sand et al., 2006; Wareing, 2014), mainly expressed in VSMCs, and small (SKCa) and intermediate (IKCa) conductance K^+^ channels, predominantly expressed in the endothelium (Sandow and Grayson, 2009; Kerr et al., 2012). In HUVECs, BKCa channel blocker iberiotoxin inhibits K^+^ currents, whereas sildenafil (Luedders et al., 2006) and insulin (Wiecha et al., 1998) activate BKCa. These last results suggest functional presence of BKCa channels in this endothelial cell type, and confirm insulin control of endothelial plasma membrane polarization. Interestingly, insulin increases L-arginine transport by modulating hCAT-1 expression and its availability at the plasma membrane (González et al., 2011), an effect that correlates with membrane hyperpolarization in HUVECs (González et al., 2004). Indeed, the bioavailability of NO and propagation of hyperpolarization from endothelial cells to VSMCs would be the main mechanisms involved in regulation of blood flow in macrocirculation and microcirculation (Figueroa and Duling, 2009). However, it is still unclear if NO generation depends on whether or not this gas induces the activity of KCa channels in the human placenta endothelium.

Other vascular tone regulators are reactive oxygen species (ROS) and nitrogen species (RNS), which constitute a family of radical and non-radical derivatives of molecular oxygen (O_2_) and nitrogen (N_2_), respectively (Klandorf and Van Dyke, 2012). Specifically, hydrogen peroxide (H_2_O_2_) and peroxynitrite (ONOO^−^) induce rapid and transient contraction or relaxation in human placental chorionic plate arteries (Mills et al., 2009). In addition, H_2_O_2_ and ONOO^−^ have higher stability than their respective precursors (Beckman and Koppenol, 1996), and may cause deleterious effects in vascular beds when insufficiently buffered/neutralized (González et al., 2011, 2015). In this regard, several reports have shown that ROS reduces NO availability and vascular relaxation in human placenta vasculature, during either healthy or pathological conditions such as preeclampsia (PE; Bernardi et al., 2008; Catarino et al., 2012), intrauterine growth restriction (IUGR; Takagi et al., 2004), and gestational diabetes mellitus (GDM; Coughlan et al., 2004).

However, it is unknown whether H_2_O_2_ might control vascular tone in the chorionic plate veins or in the placental microcirculation. It is also unknown whether insulin can regulate the vascular tone in these placental vessels. Therefore, the present study aimed to determine whether insulin attenuates the vascular response induced by H_2_O_2_ in the placental vasculature, and elucidate whether BKCa channel activity, endothelial expression, and activity of hCAT-1 are involved in this process.

## Methods

### Ethics statement

This investigation conforms to the principles outlined in the Declaration of Helsinki, and has received approval from the Ethics Committee of the Faculty of Biological Sciences of Universidad de Concepción, the Hospital Regional Guillermo Grant Benavente, Concepción Chile, and National Research Ethics System (NRES ref; 08/H1010/55), UK, and the Comisión Nacional de Investigación en Ciencia y Tecnología (CONICYT grant number 11100192, Chile). All women signed written informed consent. The maternal and newborns clinical parameters are summarized in Table 1.

**Table 1:** **Maternal and newborns clinical parameters**.

**Maternal parameters**	
Parity (median/range)	1/0–3
Maternal age (years)	27 ± 6 (18–40)
Height (m)	1.6 ± 0.1 (1.4–1.7)
Weight (kg)	81 ± 14 (55–118)
Body mass index (kg/m2)	33 ± 5 (24–44)
Fasting glucose (mg/dl)	80 ± 7 (61–93)
OGTT (mg/dL)	
Basal	80 ± 6 (61–94)
2 h	105 ± 17 (73–138)
Delivery mode	
Vaginal	43 (57%)
C-section	32 (43%)
**Newborn parameters**	
Sex (female/male)	40/35
Gestational age (weeks)	39 ± 1 (37–41)
Birth weight (kg)	3.4 ± 0.4 (2.3–4.5)
Height (cm)	51 ± 2 (44–57)
Classification	
SGA	6 (8%)
AGA	59 (79%)
LGA	10 (13%)

Women with normal pregnancies (n = 75) were included in the study (see Methods). The maternal parameters were registered before delivery. OGTT, oral glucose tolerance test; SGA, small for gestational age; AGA, appropriate for gestational age; LGA, large for gestational age. All the values are mean ± SD, unless otherwise stated.

### Human placenta and umbilical cords collection

Placentas with their umbilical cords were collected after delivery from 75 full-term normal pregnancies from the Hospital Regional Guillermo Grant Benavente in Concepción (Chile) and St. Mary's Hospital in Manchester (UK). All pregnancies were single births. The pregnant women did not smoke or consume drugs or alcohol, had no intrauterine infection or any medical or obstetrical complications, were normotensive and exhibited a normal response to the oral glucose tolerance test. They were under a normal food regimen during the whole pregnancy period and newborns were at term, born by vaginal delivery or cesarean section. Placentas were transferred in a sterile container (4°C) to the laboratory. Sections of umbilical cords (10–20 cm length) were collected into sterile 200 ml phosphate-buffered saline (PBS) solution [(mM): 130 NaCl, 2.7 KCl, 0.8 Na_2_HPO_4_, 1.4 KH_2_PO_4_ (pH 7.4, 4°C)] and used for isolation of umbilical vein endothelial cells (HUVECs) between 6–12 h after delivery.

### Cell culture

HUVECs were isolated by collagenase digestion (0.25 mg/ml Collagenase Type I from Clostridium histolyticum; Gibco Life Technologies, Grand Island, NY, USA) as previously described (González et al., 2004). In brief, cells were cultured (37°C, 5% CO_2_) up to passage 3 in medium 199 (M199) (Gibco Life Technologies, Grand Island, NY, USA) containing 5 mM D-glucose, 10% newborn calf serum (NBCS), 10% fetal calf serum (FCS), 3.2 mM L-glutamine, and 100 U/ml penicillin-streptomycin (primary culture medium, PCM). Experiments were performed on cells incubated (0–30 min) in M199 in the absence or presence of insulin (1 nM). Cell viability estimated by Trypan blue exclusion was higher than 97% (not shown). Sixteen hours prior, the experimental incubation medium was changed to sera-free M199 (González et al., 2015).

### L-arginine transport

Overall L-arginine transport (2 μCi/ml L-[^3^H]arginine (NEN, Dreieich, FRG), 0–250 μM L-arginine, 1 min, 37°C) was measured as previously described (González et al., 2015). Briefly, transport assays were performed in Krebs [in mM: 131 NaCl, 5.6 KCl, 25 NaHCO_3_, 1 NaH_2_PO_4_, 20 Hepes, 2.5 CaCl_2_, 1 MgCl_2_ (pH 7.4, 37°C)] in cells preincubated (12 h) with M199 in the absence (control) or presence (1–30 min) of insulin (1 nM). Cell monolayers were rinsed with ice-cold Krebs to terminate tracer uptake. Radioactivity in formic acid cell digests was determined by liquid scintillation counting, and uptake was corrected for D-[^3^H]mannitol (NEN) disintegrations per minute (d.p.m.) in the extracellular space. Overall transport at initial rates (i.e., linear uptake up to 1 min) was adjusted to the Michaelis-Menten hyperbola plus a nonsaturable, lineal component as described (Christensen, 1962). The maximal velocity (*V*_max_) and apparent Michaelis-Menten constant (*K*_m_) of saturable transport were calculated as described (Christensen, 1962) by this equation:

$$\begin{array}{l} v=\frac{V_{max}\cdot \left[Arg\right]}{K_{m}+\left[Arg\right]} \end{array}$$

### Immunofluorescence and confocal laser scanning microscopy

HUVECs were grown on microscope coverslips (10^6^ cells/slide) (Marienfeld GmbH & Co. KG, Lauda Königshofen, Baden-Württemberg, Germany) in PCM. Cells were incubated for 30 min in M199 in the absence or presence of 1 nM insulin (see above). Cells were then fixed in 4% paraformaldehyde (15 min), rinsed (x3) with Hanks solution [(mM): CaCl_2_ 1.26, KCl_2_ 5.37, KH_2_PO_4_ 0.44, MgSO_4_ 8.11, NaCl 136.89, Na_2_HPO_4_ 0.33, NaHCO_3_ 4.16 (37°C, pH 7.4)], permeabilized (in some experiments cells were not permeabilized) with 0.1% Triton X-100 (20 min), and blocked (1 h) with 1% BSA. Monoclonal hCAT-1 antibody (1:100) (Sigma-Aldrich, St. Louis, MO, USA) was incubated (overnight at 4°C) in PBS containing 5% BSA. Cells were washed (x3) with Hanks solution followed by incubation (1 h) with the secondary antibody, fluorescein isothiocyanate (FITC) goat anti-mouse IgG (H+L) (λexc/λem:492/520 nm) (1:2000) (Thermo Fisher Scientific, Inc., Waltham, MA, USA) in PBS containing 5% BSA. Nuclei were counterstained with Vectashield mounting media stained with 4,′6-diamidino-2-phenylindole (DAPI) (Vector Laboratories, Burlingame, CA, USA). Samples were analyzed under an Olympus IX81 microscope with a DSU spinning disk confocal system (Olympus, Tokyo, Japan). Images were obtained with a Hamamatsu ORCA-R2 camera (Hamamatsu Photonics, Hamamatsu, Japan) controlled by the Olympus XcellenceR software using a Plan Apo N 60 × 1.42 NA objective. Each sample was examined through successive 0.2 μm optical slices along the z axis. Images were analyzed using Imaris software (Switzerland), considering the fluorescence of cell volume from optical slices.

### DAF fluorescence

HUVECs were grown on microscope coverslips and intracellular NO was determined in cells incubated with insulin (1 nM, 1–30 min) and exposed (45 min, 37°C) to 10 μM of 4-amino-5-methylamino-2′,7′-difluorofluorescein (DAF-FM) (Molecular Probes, Leiden, The Netherlands). The fluorescence was observed in fixed cells by fluorescence microscopy (Olympus IX81) and the signal density was analyzed by Image J software (Java-based imaging processing program, National Institute of Health, USA).

### Isolated cotyledon perfusion

Techniques for perfusion of the placental cotyledon was that of Penfold et al. (1981), which was modified by perfusing only the fetal vascular compartment, instead of the dual perfusion model (Acevedo et al., 1995). After delivery (15–30 min), a fetal vein and artery pair on surface of chorionic plate, leading to peripheral cotyledon, was cannulated with plastic tubing. Each cotyledon was perfused with Krebs-Ringer solution at a constant flow rate (7 mL/min), maintained with oxygen levels similar to physiological conditions for placental vessels *in situ*. The perfusion pressure was continuously monitored and the viability of preparation was controlled as previously described (Acevedo et al., 1999).

### Wire myography

Chorionic plate veins, identified as branches of the umbilical vein, were dissected from biopsies and placed in an ice-cold physiological saline solution (PSS). Veins were mounted on a myograph (610 M; Danish Myotechnology, Aarhus, Denmark) and normalized to 0.9 L_5.1kPa_as described (Mills et al., 2009). Vessels were bathed in PSS and maintained with oxygen levels similar to physiological conditions for chorionic vessels *in situ* (Mills et al., 2009). After the vein rings were stabilized for isometric force measurements with optimal diameter (~310 μm), the maximal active response was determined with modified PSS containing 90 mM KCl. Two different protocols were designed to determine the effects of insulin consisting of incubation of pre-constricted (U46619) veins with the hormone (in the presence of inhibitors); or preincubation (30 min) with insulin (in the presence of inhibitors) prior to U46619 (10^−10^–10^−5^ M) or H_2_O_2_ (10^−5^–10^−3^ M) exposure.

### Pharmacological agents

General chemicals and pharmacological agents were purchased from Sigma-Aldrich, St. Louis, MO, USA, including; insulin (1–10 nM), H_2_O_2_(0.01–1 mM), tetraethylammonium (1 mM) (KCa channels inhibitor), iberiotoxin (100 nM) (BKCa channels inhibitor), genistein (50 μM) (tyrosine kinases inhibitor) and wortmannin (30 nM) (phosphatidylinositol 3-kinase inhibitor). U46619 (0.0001–1 μM; thromboxane A2 analog) was obtained from Tocris Bioscience, Bristol, UK.

### Statistical analysis

Values are mean ± S.E.M., where *n* indicates the number of different cell cultures (three to four replicates). Comparisons between two or more groups were performed by means of Student's unpaired *t*-test and analysis of variance (ANOVA), respectively. If the ANOVA demonstrated a significant interaction between variables, *post-hoc* analyses were performed by the multiple-comparison Bonferroni correction test. Values of *p* < 0.05 were considered statistically significant.

## Results

### Insulin increases the L-arginine/NO pathway in HUVECs

**Figure 1:**
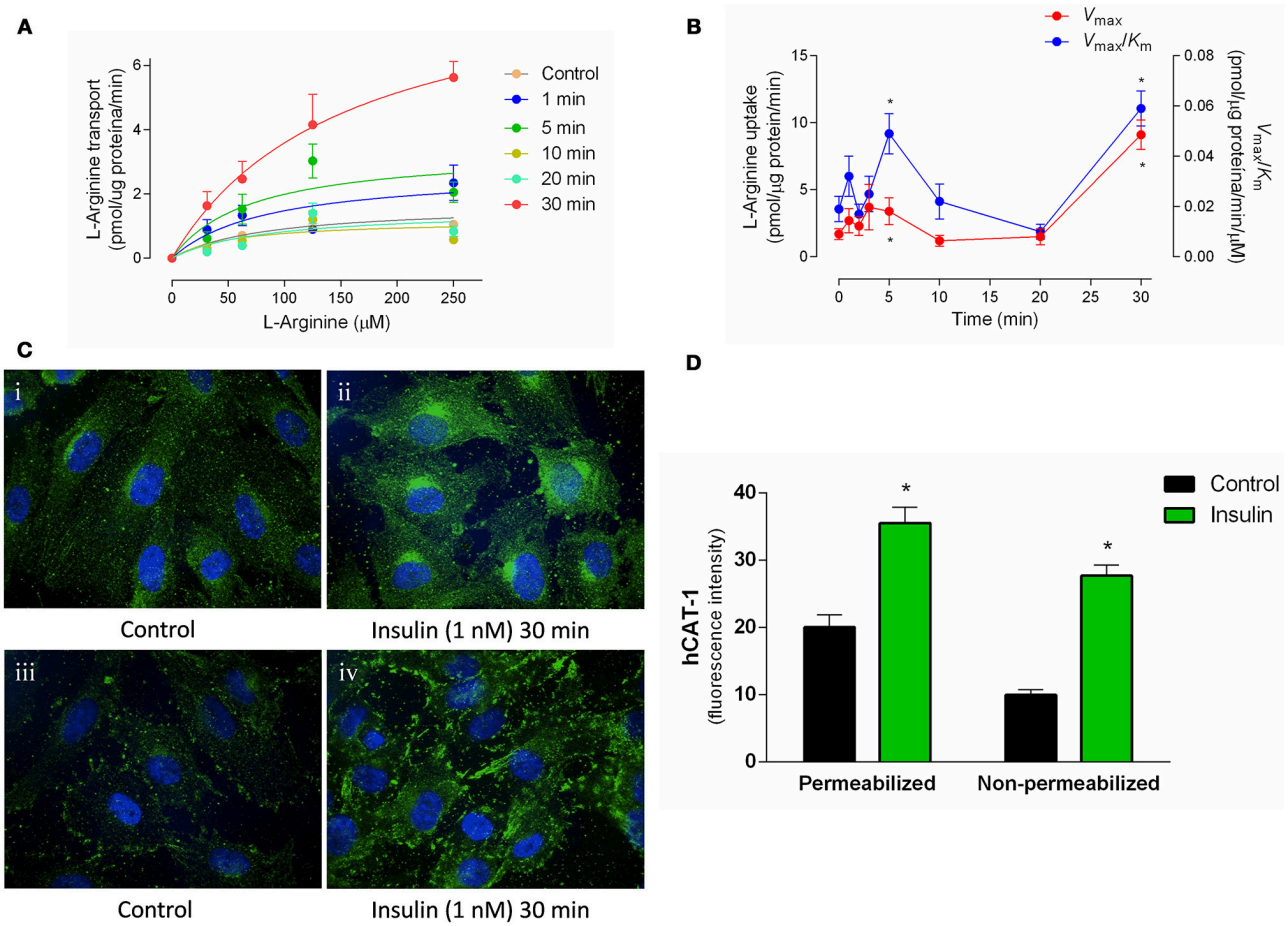
**Insulin induces rapid increases in the hCAT-1 activity**. L-Arginine transport (0–250 μmol/L L-arginine, 2 μCi/mL L-[^3^H]arginine, 1 min, 37°C) and hCAT-1 expression were determined in HUVECs pre-incubated (1–30 min) in medium 199 in absence (control) or presence of 1 nM insulin. Saturable transport was adjusted to Michaelis-Menten kinetic curve **(A)** and maximal velocity (*V*_max_) and maximal transport capacity (*V*_max_/*K*_m_) values were plotted and calculated from experimental data **(B)**. hCAT-1 expression was determined through immunocytochemistry (green fluorescence) in permeabilized **(Ci; Cii)** or non-permeabilized **(Ciii; Civ)** cells. Control cells are in the absence of insulin. Images were obtained with 60x magnification in confocal microscopy. **(D)** Mean fluorescence intensity was determined based on cells volume of three different fields of each experiment and values in y-axis are presented as arbitrary units. ^*^P < 0.05 vs. values in the absence of insulin. In **(A,B)**, values are mean ± S.E.M. (*n* = 12–15). In **(C)**, images are representative of three different cell cultures and graph **(D)** shows mean ± S.E.M. (*n* = 3).

**Figure 2:**
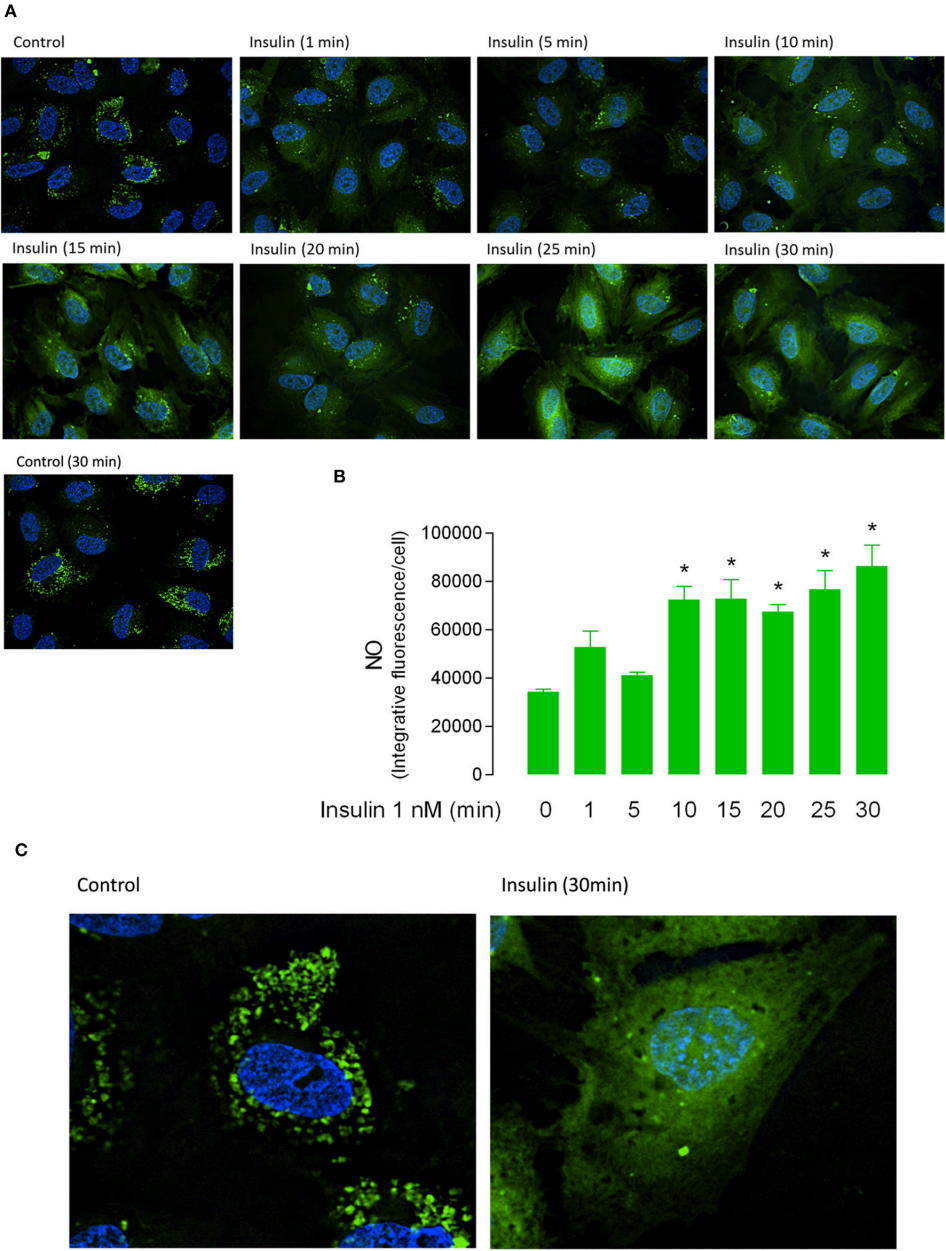
**Short-term incubation with insulin increases the nitric oxide synthesis in fetal endothelium**. Nitric oxide (NO) in HUVEC incubated in absence (Control 0, 30 min) or presence of 1 nM insulin (1–30 min). **(A)** Green fluorescence was observed through confocal microscopy in cells preloaded (30 min) with 4-amino-5-methylamino-2′,7′-difluorofluorescein (DAF-FM). **(B)** The integrative fluorescence per image was determined using Image J software and normalized by number of cells. **(C)** Magnification of fluorescence in control and insulin-treated cells, shown in **(A)**. The images **(A,C)** are representative of triplicates from three different cell cultures.^*^P < 0.05 vs. values in the absence of insulin (*n* = 3).

Apparent *K*_m_ was maintained in a range between 54 ± 16 to 153 ± 18 μM (Figure 1A, Table 2) in all experimental conditions. Insulin increased the *V*_max_ of L-arginine transport with maximal effect (5.4 ± 0.9-fold) after 30 min of treatment and lower but significant (*p* < 0.005) increases after 3 min (2.2 ± 0.8-fold) and 5 min (2 ± 0.5-fold) of incubation (Figures 1A,B, Table 2). No changes in *V*_max_ were detected after 10 or 20 min of treatment with insulin. The *V*_max_/*K*_m_ was significantly increased 2.6 ± 0.8 and 2.5 ± 0.9-fold after 5 min and 30 min incubation with 1 nM insulin, respectively (Figure 1B, Table 2). Insulin (1 nM, 30 min) increased hCAT-1–associated fluorescence in permeabilized (1.8 ± 0.3-fold) and non-permeabilized (2.8 ± 0.3-fold) cells, compared to control (Figure 1C). The fold of increase induced by insulin in hCAT-1-associated fluorescence is 55% higher (2.8 vs. 1.8-fold) in non-permeabilized cells compared with permeabilized cells. From total fluorescence, in insulin-treated cells the 78% (27.8 vs. 35.6 arbitrary units of fluorescence) correspond to hCAT-1 expression on cell surface, meanwhile this percentage decreased until 50% (10 vs. 20 arbitrary units of fluorescence) in control cells (Figure 1D). Insulin also increased NO levels (Figure 2A) with maximal effect (2.5 ± 0.2-fold) (Figure 2B) after 30 min of incubation. No changes were detected in control cells incubated by 30 min in medium without insulin (Figure 2A). The basal level of DAF fluorescence in HUVECs was largely detected in a vesicular-like form; however, in cells incubated with insulin, the fluorescence was diffused throughout the cytoplasm (Figure 2C).

**Table 2:** **Effect of insulin on L-arginine transport in HUVECs**.

	**L-Arginine transport kinetics**		
	***V_max_* (pmol/ μg protein/min)**	***K_m_* (μM)**	***V_max_*/*K_m_* (pmol/ μg protein/min/(μM))**
Control	1.7±0.4	88±23	0.019±0.005
Insulin 1 min	2.7±0.9	84±18	0.032±0.008
Insulin 2 min	2.3±0.7	130±22	0.017±0.004
Insulin 3 min	3.7±1.7^*^	145±28	0.025±0.007
Insulin 5 min	3.4±1.0^*^	70±12	0.049±0.018^*^
Insulin 10 min	1.2±0.4	54±16	0.022±0.007
Insulin 20 min	1.5±0.6	84±12	0.010±0.003
Insulin 30 min	9.1±1.1^*^	153±18	0.059±0.007^*^

*Kinetics of saturable L-arginine transport (0–250 μM L-arginine, 2 μCi/ml L-[^3^H]arginine, 1 min, 37°C) was measured in HUVECs exposed (1–30 min) to Krebs solution in absence (Control), or containing 1 nM insulin (see Methods). Maximal transport capacity was determined by the V_max_/K_m_ ratio for L-arginine transport*.

* *P < 0.05 versus control. Values are means ± SEM (n = 10–12)*.

### Placental relaxation induced by insulin

**Figure 3:**
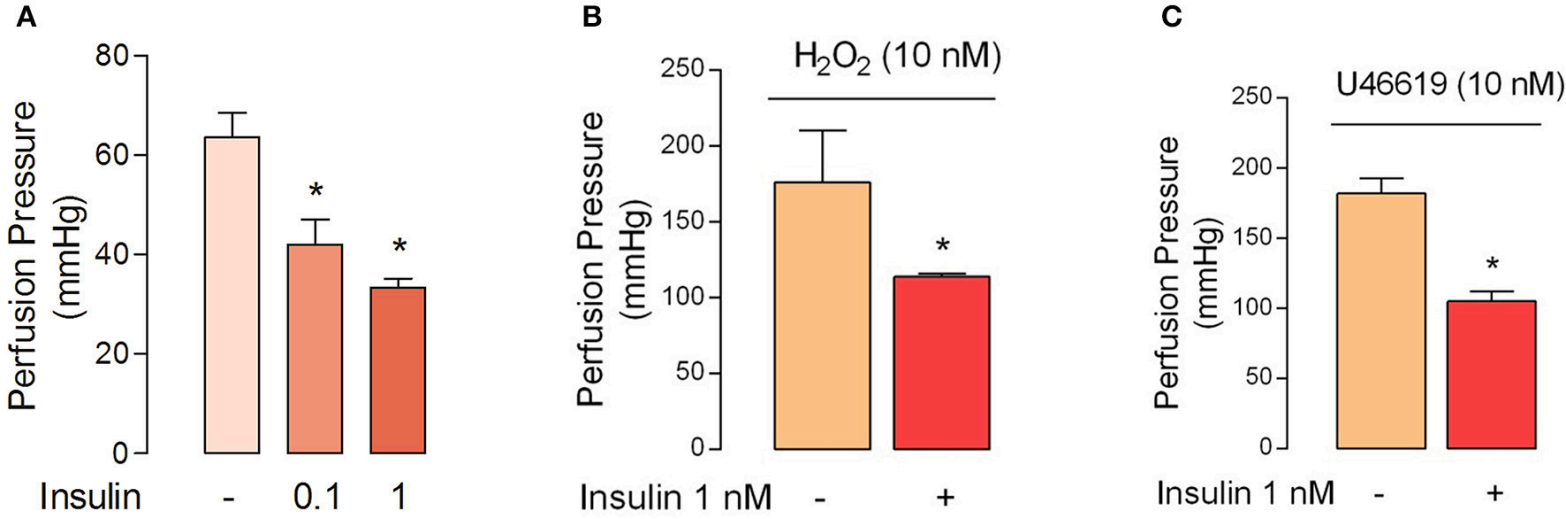
**Insulin reduces the perfusion pressure in fetal-side circulation of isolated cotyledon from human placenta. (A)** Perfusion pressure was measured in absence (−) or presence of insulin (30 min) in placentae, which have a mean basal perfusion pressure of 64 ± 5 mmHg. **(B,C)** Perfusion pressure was measured in placentae perfused (30 min) with Krebs solution in absence (−) or presence (+) of insulin and later perfused with H_2_O_2_ **(B)** or U46619 **(C)**. ^*^P < 0.05 vs. basal conditions in the absence of insulin. Values are mean ± S.E.M. (*n* = 5).

**Figure 4:**
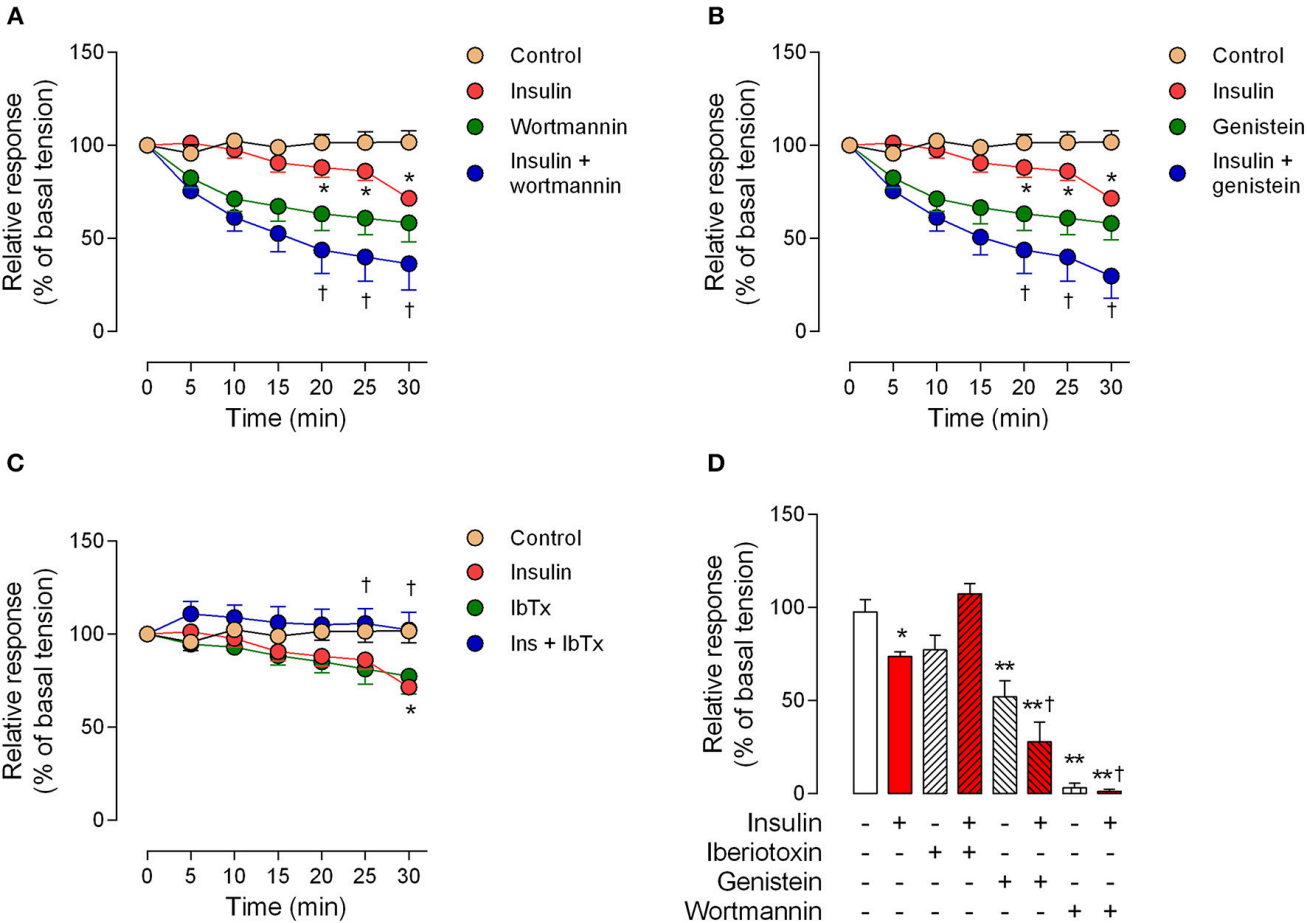
**Relaxation induced by insulin is blocked by iberiotoxin**. Response of human chorionic vein rings pre-constricted with U46619 and exposed to 10 nM insulin is showed in presence of 30 nM wortmannin **(A)**, 50 μM genistein **(B)** or 100 nM iberiotoxin **(C)**. Maximal responses are shown in **(D)**, as a percentage fraction of the initial vessel response to KCl (see Methods). ^*^P < 0.05 vs. non-treated (control) vessels. ^**^P < 0.01 vs. non-treated (control) vessels. ^†^*P* < 0.05 vs. values in insulin-treated vessels. Values are mean ± S.E.M. (*n* = 5–10).

**Figure 5:**
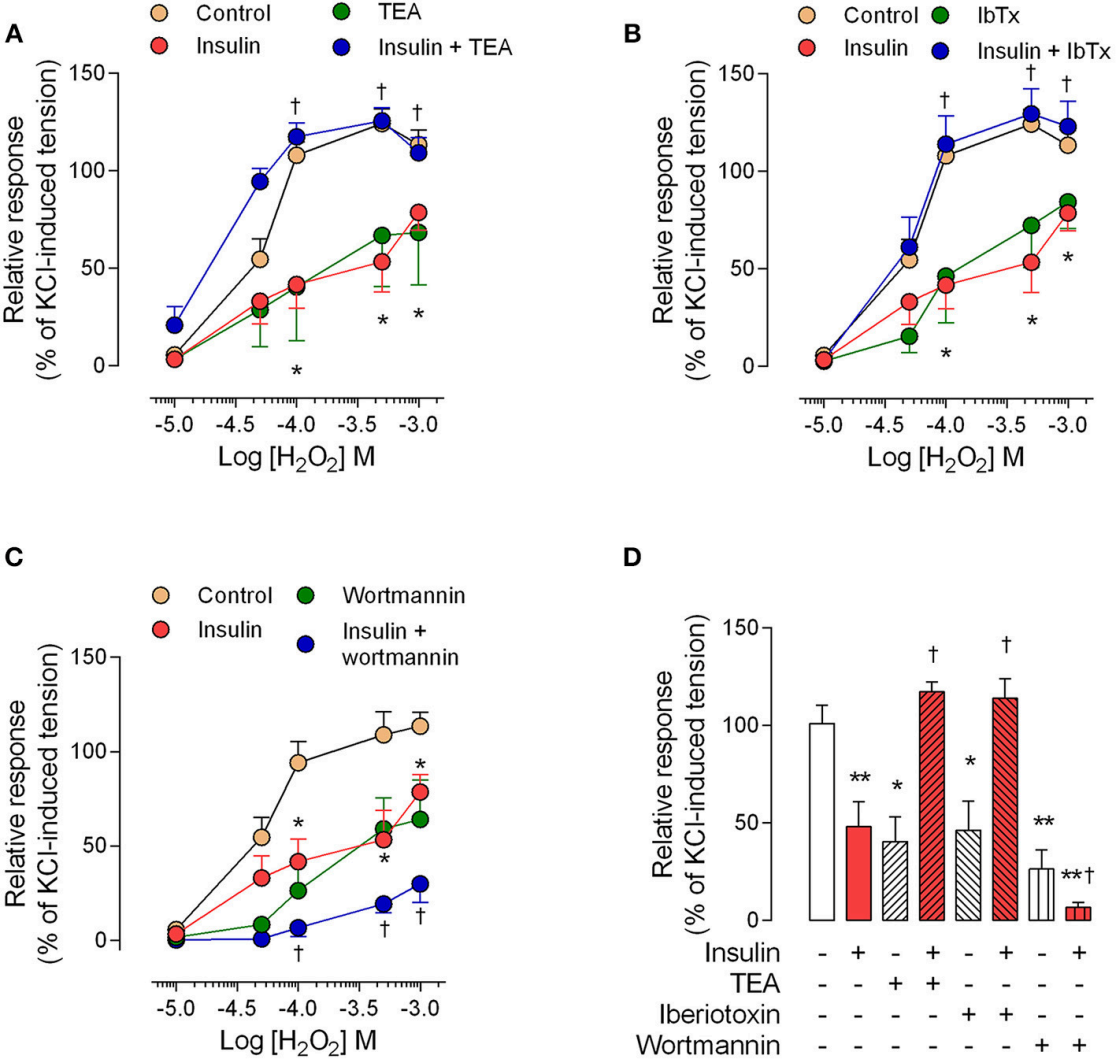
**The effect of pre-incubation with insulin on H_2_O_2_ constriction is dependent on BKCa channels**. Chorionic vein rings were pre-incubated (30 min, 5%CO_2_, 37°C) in absence (control) or presence of 10 nM insulin and/or 1 mM tetraethylammonium (TEA; **A**), 100 nM iberiotoxin (IbTx; **B**), or 30 nM wortmannin **(C)**. After these treatments, vessels were exposed to hydrogen peroxide (H_2_O_2_). In **(D)** the effects are shown of treatments on maximal constriction induced by 100 μM (1 × 10^−4^ M) H_2_O_2_. ^*^P < 0.05, ^**^P < 0.01 vs. values in the absence of insulin. ^†^*P* < 0.05 vs. values in insulin treated vessels. Values are mean ± S.E.M. (*n* = 5–7).

**Figure 6:**
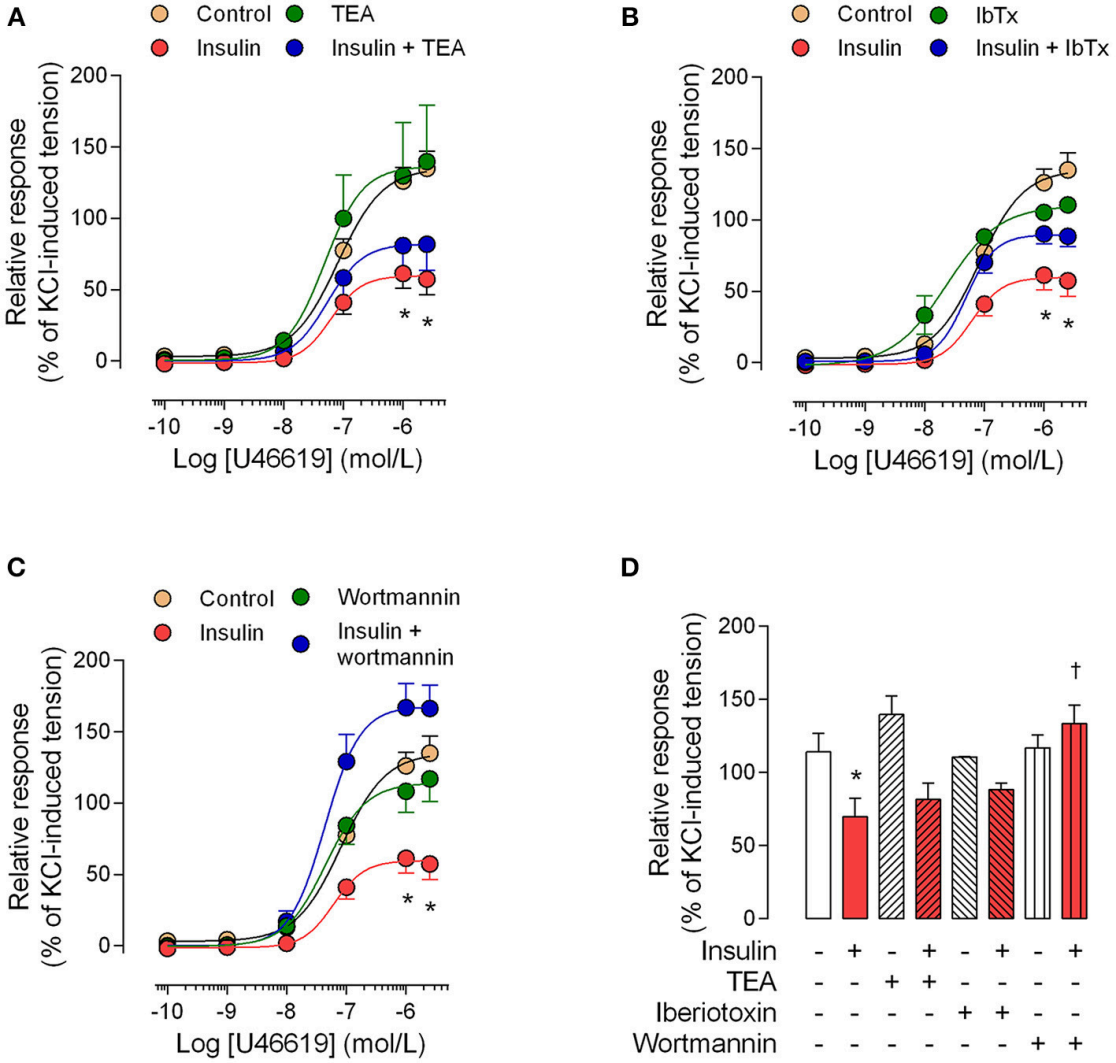
**The effect of pre-incubation with insulin on U46619 constriction is dependent on PI3K**. Chorionic vein rings were pre-incubated (30 min, 5%CO_2_, 37°C) in absence (control) or presence of 10 nM insulin and/or 1 mM tetraethylammonium (TEA; **A**), 100 nM iberiotoxin (IbTx; **B**) or 30 nM wortmannin **(C)**. After these treatments, vessels were exposed to U46619. In **(D)**, the effects are shown of treatments on maximal constriction induced by 1 μM (1 × 10^−6^ M) U46619. ^*^P < 0.05 vs. values in the absence of insulin. ^†^*P* < 0.05 vs. values in insulin-treated vessels. Values are mean ± S.E.M. (*n* = 5–7).

The perfusion pressure in isolated cotyledon of placenta decreased from 64 ± 5 mmHg to 42 ± 5 mmHg and 33 ± 2 mmHg using 0.1 and 1 nM of insulin, respectively (Figure 3A); without changes in the flow (not shown). In the same preparation, both H_2_O_2_(2.5 ± 0.4-fold) and U46619 (3.4 ± 0.5-fold) increased perfusion pressure, effects attenuated by preincubation with insulin (1 nM; Figures 3B,C). In chorionic veins preconstricted with U46619, insulin (10 nM) caused 29 ± 2% of relaxation after 30 min of treatment (Figure 4). Coincubation with wortmannin (Figure 4A) or genistein (Figure 4B) did not block insulin's vasodilator effect. Indeed, vessels exposed to tyrosine kinases inhibitors showed relaxation of 61 ± 10 and 52 ± 5% with wortmannin and genistein, respectively (Figure 4D). In the presence of insulin, maximal relaxation caused by wortmannin or genistein was further increased (73 ± 15 and 71 ± 9%, respectively; Figure 4D). The effect of insulin on relaxation caused in preconstricted chorionic vein was blocked by coincubation with iberiotoxin (Figure 4C) meanwhile the BKCa inhibitor induced a maximal relaxation of 21 ± 2% in absence of insulin (Figure 4D). Using a different protocol (pre-incubation of 30 min with insulin), insulin (10 nM) decreased by 49 ± 5% the constriction induced by 100 μM H_2_O_2_(Figure 5A). Reduced vasoconstriction caused by insulin was abolished by coincubation with tetraethylammonium (TEA, Figure 5A) or IbTx (Figure 5B), but not with wortmannin (Figure 5C). Preincubation with wortmannin potentiated the effect of insulin on H_2_O_2_ constriction in 40 ± 8% (Figure 5C). Insulin, TEA, IbTx and wortmannin decreased the constriction caused by H_2_O_2_by 53 ± 8, 60 ± 11, 55 ± 10, and 74 ± 15%, respectively. Moreover, the combination of insulin and wortmannin exhibited the highest inhibition on H_2_O_2_ constriction (93 ± 16%; Figure 5D). In addition, the maximal contractile response to U46619 was reduced 56 ± 4% following preincubation with insulin. Meanwhile, the half-maximal effective concentration (*EC*_50_) of U46619 was reduced from 81 ± 12 nM to 61 ± 6 nM in insulin-treated veins (Figure 6). However, when vessels were constricted with U46619, insulin–associated relaxation was unaltered by TEA or IbTx (Figures 6A,B), but was blocked by wortmannin (Figure 6C).

## Discussion

The mechanism previously reported for vascular properties of insulin involves higher expression and activity of eNOS in HUVECs (Montagnani et al., 2002; González et al., 2004), human aortic coronary endothelial cells (HAECs; Federici et al., 2002) and bovine aortic endothelial cells (BAECs; Kuboki et al., 2000). The effect of long-term insulin incubation (several hours) on eNOS activity is known to be dependent on PI3K signaling (Montagnani et al., 2002; González et al., 2011). However, mechanisms underlying a rapid stimulation of vasodilatation induced by insulin (González et al., 2011) are still unclear, and have been associated with NO–mediated blood flow in healthy subjects (Scherrer et al., 1994; Steinberg et al., 1994; Lind et al., 2002). In this study, we demonstrated that insulin induces relaxation in pre-constricted vessels and attenuates constriction (when hormone is pre-incubated) in a mechanism mediated by BKCa channel activity and related to increased L-arginine transport and endothelial NO synthesis.

### Mechanism of relaxation induced by insulin

Insulin caused 42% relaxation in pre-constricted human umbilical vein (González et al., 2011). The *EC*_50_ of insulin was 1.8 ± 0.2 nM and the effect was abolished by preincubation with *N*-ethylmaleimide (NEM) and L-lysine, both competitive inhibitors of transport system *y*^+^ for cationic amino acid (González et al., 2011). Now, we confirmed that insulin induced relaxation in chorionic plate veins and in fetal-side of placental vascular bed, decreasing the vasoconstriction induced by U46619 and H_2_O_2_, stimuli that had previously been shown to have vasoconstrictor effects in chorionic plate arteries (Beckman and Koppenol, 1996; Hayward et al., 2013).

Relaxation induced by insulin in placental vessels was related to L-arginine/NO pathway in HUVECs. Previously, insulin has been noted to increase L-citrulline synthesis and L-arginine transport using concentrations between 0.1 and 10 nM in long-term (8 h) incubation assays, via a mechanism that involves high expression of hCAT-1 (Sobrevia et al., 1996; González et al., 2004). In isolated umbilical vein rings, insulin induced relaxation after 30 min of incubation, an effect disrupted by co-incubation with L-lysine, NEM or by endothelial denudation (González et al., 2011). This study confirms the effect of insulin in human placenta, and also suggests that this phenomenon is related to high NO synthesis, L-arginine transport and higher abundance of hCAT-1 in HUVECs. Regarding cell surface expression of hCAT-1, it has been determined that activation of protein kinase C (PKC) reduces cell surface expression of hCAT-1 and L-arginine transport in *Xenopus laevis* oocytes and U373MG glioblastoma cells (Rotmann et al., 2004a,b), reinforcing the idea that a rapid mobilization of hCAT-1 from plasma membrane to cytoplasm reduces L-arginine transport. Moreover, our work suggests that high *V*_max_ of L-arginine transport mediated by insulin is associated with mobilization of hCAT-1 from cytoplasm to cell surface in HUVECs considering that insulin treatment results in a larger percentage of the total fluorescence from hCAT-1 expression on the surface of the cells. Further studies are necessary to determine the functional co-localization of hCAT-1 with relevant proteins for endothelial cell function like caveolin 1 or eNOS, as has been shown in porcine aortic endothelial cells (PAECs) (McDonald et al., 1997) or baby hamster kidney (BHK) cells (Lu and Silver, 2000).

One of the main mechanisms triggered by insulin is related to activation of PI3K in the human endothelium (González et al., 2004; Muniyappa et al., 2008). However, our results showed that inhibition of PI3K induces vessel relaxation; which was potentiated by insulin. Similarly, genistein induced relaxation in an endothelium-independent mechanism in rat aorta pre-constricted by sodium fluoride (NaF). In addition, when vessels were pre-treated with genistein, there was a decrease in constriction induced by U46619 (Je and Sohn, 2009) and an increase of eNOS phosphorylation in serine 1179 after 10–30 min of incubation via a protein kinase A (PKA)-dependent mechanism in BAEC (Liu et al., 2004). In the endothelial cell line ECV-304, the pre-incubation with genistein improved cell viability and reversed apoptosis induced by H_2_O_2_, which was associated with enhanced antioxidant capacity (Jin et al., 2015). On the other hand, the activity of protein kinase B/*Akt* plays a central role in the PI3K-dependent activation of eNOS catalyzing the phosphorylation of serine 1179 and serine 617, increasing the sensitivity of eNOS by Ca^2+^/calmodulin (CaM) complex (Tran et al., 2009). Previously, it has been shown that 100 nM insulin induces the phosphorylation of PKB/Akt after 2 or 10 min of treatment in HUVEC, increasing the eNOS activity in a mechanism dependent on insulin receptor substrate 1 (IRS1; Federici et al., 2004). With these evidences accounted for, our results show that inhibition of tyrosine kinases induces relaxation in pre-constricted placental vessels and, more importantly, the relaxation induced by insulin could be independent of PI3K pathway. It will still be important to explore the effects of insulin in PKB/*Akt* activity and CaM-dependent activation of eNOS, especially if the insulin-induced relaxation is associated with activity of K^+^ channels activated by Ca^2+^. In regard to this association, recently it has been shown that genistein, in combination with magnesium, induces relaxation in rat mesenteric arteries in a mechanism dependent on eNOS activity (blocked by L-NAME) and associated with high BKCa currents in rat mesenteric smooth muscle cells (Sun et al., 2015). These results suggest that genistein has a dual effect both in endothelial cells and VSMCs. In our experiments it is possible that the effects of genistein in the chorionic veins were not due to inhibition of protein kinases mainly, because we cannot discard the activation of BKCa by genistein directly in chorionic vein smooth muscle cells.

### Role of potassium channels activity

Using hippocampal neurons, O'Malley and Harvey (2004) showed that insulin (10 nM) increased (~3.8-fold) the mean channel activity (Nf*P*_o_) of BKCa after 15 min incubation in the bath solution. Meanwhile, in similar experiments but via patch pipette solution, the effect was faster (2–8 min post-insulin). Authors also showed that insulin increased the mean open time (τ_o_) of BKCa from ~0.76 ms (at 2–4 min) to ~2.01 ms (at 15–17 min). Similar effects were observed in HEK293 cells expressing h*Slo* (pore-forming α subunit of BKCa channel) and direct activation of BKCa channels through application of its selective channel opener, NS-1619 (O'Malley and Harvey, 2004). Similar to our results, this last study showed that the effect of insulin on BKCa activity is mediated by a mechanism independent of PI3K (but dependent of Ras/Raf/MEK/ERK pathway). Therefore, our study shows that the effects of insulin on placental vasculature are independent of PI3K activity, but mainly dependent of BKCa activity. We acknowledge that further experiments are required in order to elucidate underling intracellular pathway linked with this effect of insulin on BKCa channels, including potential participation of MAPK pathway.

Regarding the KCa expression and activity in human placenta, in 2006 Wareing et al. reported mRNA expression of BKCa in placental arteries and veins, showing high basal expression in arteries, suggesting a role of BKCa in fetoplacental relaxation induced by NO (Wareing et al., 2006). More recently, in chorionic plate artery smooth muscle cells (CPASMCs), the incubation with TEA and iberiotoxin demonstrated that BKCa are the main channels responsible for outward currents in CPASMCs (Brereton et al., 2013). Importantly, immunohistochemistry assays in placental tissue, have demonstrated a strong expression of BKCa in endothelium with similar localization of endothelial cell marker CD31 (Sand et al., 2006). In our study, we cannot discern if BKCa expression/activity is more important in endothelium or VSMCs, but the relaxation induced by TEA and iberiotoxin reveals a role of BKCa in the vascular tone regulation of placenta. In rabbit basilar artery, similar pre-incubation (30 min) with iberiotoxin and TEA reduced the vascular tone induced by sodium acetate (Cho et al., 2007). This result is similar to our finding about the reduction of vascular tone induced by H_2_O_2_; in both cases the mechanism of constriction is not via a receptor-mediated signaling pathway. The mechanism of vascular tone modulation by H_2_O_2_ in placenta is still unclear, but previous report of Mills showed a transient constriction induced by H_2_O_2_ in chorionic plate arteries, reversed by catalase (Mills et al., 2009). In umbilical artery, similar concentration (10–100 μM) of H_2_O_2_ enhanced the tension induced by prostanglandin F2α without change in sensitivity to calcium chloride (Okatani et al., 1997). The short half-life and rapid conversion of H_2_O_2_ to other reactive species, like hydroxyl radical (^•^OH) through Fenton reaction in presence of Fe^2+^ or Haber-Weiss reaction in presence of ${\text{O}}_{2}^{-}$ (MacFarlane et al., 2008), suggest a potential role of ^•^OH in H_2_O_2_-induced constriction in placental vessels. At moment, we still cannot explain with direct evidence the effect of TEA or iberiotoxin on H_2_O_2_-induced constriction, but we speculate that the alteration in vascular homeostasis after 30 min of BKCa inhibition could reduce the constriction associated with oxidative stress. Although in our study we did not determine the changes in plasma membrane potential, is possible that a change of resting potential (both in endothelium and VSMCs) induced by iberiotoxin alters the vascular response to oxidative stress.

Related to the connection between the *ex vivo* and *in vitro* results, previously it has been demonstrated that HUVECs express Ca^2+^-activated potassium currents blocked by iberiotoxin (Wiecha et al., 1998; Watanapa et al., 2012). More importantly, 0.6 nM (100 μUI/ml) insulin increased the open-state probability (NPo) of BKCa after 3 min incubation in this cell type (Wiecha et al., 1998), in a similar fashion that 10 nM insulin increased the activity of BKCa in O'Malley and Harvey study. A different stimulus, quercetin, induces hyperpolarization, high concentration of intracellular Ca^2+^, cGMP synthesis and reduction of proliferation of HUVECs, and each of these effects are blocked when the cells are incubated with iberiotoxin (Kuhlmann et al., 2005). In addition, HUVECs treated with plasma samples from preeclamptic pregnancies exhibit a higher fraction of cells expressing outward currents associated with KCa channels, showing a compensatory mechanism attributed to some factors secreted in preeclampsia (Watanapa et al., 2012). These evidences allow us to propose that the insulin signaling induces the activation of KCa channels, changing the plasma membrane polarity for activation of hCAT-1 reflected in higher *V*_max_ for L-arginine transport and, finally, higher NO synthesis. In this regard, a study published by Kavanaugh showed that the influx of L-arginine is increased by membrane hyperpolarization in *Xenopus laevis* oocytes expressing CAT-1 (Kavanaugh, 1993). Oppositely, the depolarization induced by increased extracellular concentration of K^+^ reduced the L-arginine transport in HUVECs (Sobrevia et al., 1995). Also in *Xenopus laevis* oocytes expressing hCAT-1, the incubation (6 h) with high concentration of K^+^ reduced the intracellular accumulation of L-[^3^H]arginine (Rotmann et al., 2004a). These findings show the dependency between hCAT-1 activity and voltage of the plasma membrane, but further studies are necessary to establish direct evidence of regulation of hCAT-1 activity through membrane hyperpolarization in placental endothelial cells.

Also, it is important to note that previously it has been shown that insulin evokes hyperpolarization (from −65.5 ± 0.4 to −82.3 ± 0.4 mV) and high intracellular concentration of Ca^2+^ (from 40 ± 3 nM to 372 ± 29 nM) in HUVECs (González et al., 2004). Although the mechanism of intracellular Ca^2+^ regulation by insulin in endothelium still is not clear, other agonists or stimuli that activate eNOS have been studied. VEGF-A and shear stress increase the activity of eNOS and NO synthesis in a mechanism dependent of enhancement of Ca^2+^ (Devika and Jaffar Ali, 2013). Importantly, Anaya et al., showed that the Ca^2+^ mobilization and NO availability (detected with DAF) were reduced in intact endothelium from umbilical vein isolated of GDM samples, without changes in the protein abundance of eNOS. Additionally, in HUVECs isolated from GDM, the response of Ca^2+^ mobilization to ATP is lower than control cells (Anaya et al., 2015). From these data, we suggest that the effects of insulin on L-arginine/NO pathway in HUVECs and BKCa-dependent relaxation in chorionic vein, reported in this study, could be significantly altered in GDM due to reduced capacity of endothelial cells for increased Ca^2+^ mobilization in response to some agonists.

In conclusion, we found that insulin induces a rapid relaxation in placental vascular bed through a mechanism associated with high activity of BKCa channels and L-arginine/NO pathways in endothelial cells.

## Author contributions

LC, AS, CV, DG, PC, EB, AH: Experimental work and first analysis of results. This article is a result of different undergraduate projects executed by these authors. SR: Experimental work, supervision and technical support in Vascular Physiology Laboratory. MC: Collection of informed consent and verification of clinical data of pregnants. EP: Acquisition of images from confocal microscopy. CR, JV: Collaboration with fluorescence results analysis. VG: Collaboration with discussion about the clinical implications of results. LZ: Collaboration in discussion related with calcium-activated potassium channels. CE, LS: Collaboration with discussion about the mollecular mechanisms involved. MW: Collaboration with the execution and analysis of wire myography experiments. MG: Supervision of experimental work, analysis of results, coordination and main discussion of the article.

### Conflict of interest statement

The authors declare that the research was conducted in the absence of any commercial or financial relationships that could be construed as a potential conflict of interest. The reviewer LM and handling Editor declared their shared affiliation, and the handling Editor states that the process nevertheless met the standards of a fair and objective review.

## Abbreviations

BAECs: Bovine aortic endothelial cells
BHK: Baby hamster kidney cells
BKCa: Large conductance calcium-activated potassium channels
eNOS: Endothelial nitric oxide synthase
GDM: Gestational diabetes mellitus
HAECs: Human aortic endothelial cells
hCAT-1: Human cationic amino acid transporter 1
HUVECs: Human umbilical vein endothelial cells
IbTx: Iberiotoxin
IUGR: Intrauterine growth restriction
KCa: Calcium-activated potassium channels
MAPK: Mitogen-activated protein kinases
NEM: N-ethylmaleimide
NO: Nitric oxide
PAECs: Porcine aortic endothelial cells
PE: Preeclampsia
PI3K: Phosphatidylinositol 3-phosphate
PKC: Protein kinase C
RNS: Reactive nitrogen species
ROS: Reactive oxygen species
SLC7A1: Solute carrier family 7 type 1
TEA: Tetraethylammonium
VSMCs: Vascular smooth muscle cells.
